# Quality of life of survivors of malignant pleural mesothelioma in Japan: a cross sectional study

**DOI:** 10.1186/s12885-018-4293-x

**Published:** 2018-03-27

**Authors:** Yasuko Nagamatsu, Isao Oze, Keisuke Aoe, Katsuyuki Hotta, Katsuya Kato, Junko Nakagawa, Keiko Hara, Takumi Kishimoto, Nobukazu Fujimoto

**Affiliations:** 1 Graduate School of Nursing Science, St. Luke’s International University, 10-1 Akashicho, Chuo-ku, Tokyo, 1040044 Japan; 2 Division of Molecular and Clinical Epidemiology, Aichi Cancer Center Research Institute, 1-1 Kanokoden, Chigusa-ku, Nagoya, 4648681 Japan; 3 National Hospital Organization Yamaguchi-Ube Medical Center, Department of Medical Oncology, 685 Higashikiwa, Ube, 7550241 Japan; 4 Center for Innovative Clinical Medicine, Okayama University Hospital, 2-5-1 Shikatacho, Okayama, 7008558 Japan; 5 Department of Radiology, Kawasaki General Medical Center, 2-6-1 Nakasange, Okayama, 7008505 Japan; 6 Department of Nursing, Okayama Rosai Hospital, 1-10-25 Chikkomidorimachi, Okayama, 7028055 Japan; 7 Department of Medicine, Okayama Rosai Hospital, 1-10-25 Chikkomidorimachi, Okayama, 7028055 Japan; 8 Department of Medical Oncology, Okayama Rosai Hospital, 1-10-25 Chikkomidorimachi, Okayama, 7028055 Japan

**Keywords:** Asbestos, CoQoLo, Mesothelioma, Palliative care, Quality of life, Questionnaire

## Abstract

**Background:**

Previous studies have indicated that people with malignant pleural mesothelioma (MPM) have a poor quality of life (QOL); however, information about the QOL of people with MPM in Japan is anecdotal. The aims of this study were to investigate the QOL of survivors of MPM in Japan and to determine the factors that correlate with their QOL.

**Methods:**

This was a cross sectional study. The included patients were those diagnosed with MPM in Japan. We created a self-administered questionnaire consisting of 64 questions. The questionnaires were sent to hospitals and patient advocacy groups, distributed to the patients, completed, and sent back to the researchers by postal mail. QOL was assessed with the European Organization for Research and Treatment of Cancer 16 questionnaire (QLQ) and the short version of the core domains of the Comprehensive Quality of Life Outcome questionnaire (CoQoLo).

**Results:**

In total, 133 questionnaires were collected. The QLQ assessments demonstrated that the survivors of MPM most frequently complained of fatigue, pain, sleep disturbances, and dyspnea. The symptom scales were acceptable, but the functional scales were significantly poorer for the patients with poor performance statuses (PSs). The short CoQoLo assessment was very unfavorable for ‘Being free from physical pain.’ Being a long-term survivor and a survivor with a poor PS were significantly correlated with poor global health status.

**Conclusions:**

Survivors of MPM have impaired function, a variety of symptoms, and lower QOL. Survivors of MPM, even those in good physical condition, need broad support.

## Background

The World Health Organization (WHO) reported that 107,000 people die from occupational exposure to asbestos each year, and the WHO advocates for the elimination of asbestos-related diseases [1, 2]. Mesothelioma is a rare malignancy caused by asbestos exposure that affects the pleura, peritoneum, and pericardium [3]. Malignant pleural mesothelioma (MPM), which is the most common mesothelioma, is almost always fatal [4]. The overall median survival time and 2-year survival rate of patients with resectable disease, who have undergone trimodal treatment composed of induction chemotherapy followed by extrapleural pneumonectomy and postoperative radiation therapy, are 19.9 months and 42.9%, respectively [5], and the median overall survival of patients with advanced surgically unresectable disease who received cisplatin and pemetrexed is approximately 12 months [6]. Additionally, MPM causes debilitating physical symptoms, such as pain, dyspnea, fatigue, and loss of appetite [7, 8]. The British Thoracic Society Standards of Care Committee recommends that palliative care and symptom control be central to any management plan for mesothelioma patients [9]. Recently, maintaining patients’ quality of life (QOL) has become more important in the treatment of MPM because of its poor prognosis. The Australian guidelines were developed by employing questions about the QOL of the patient, interventions, comparisons, and outcomes [4]. The QOL has been assessed in studies of treatments for MPM, such as chemotherapy [10], pleurectomy [11], and extrapleural pneumonectomy [12]. There are previous reports that MPM impairs the QOL of patients and their care givers [13, 14]. Kao et al. reported that health-related QOL is associated with survival in MPM patients [15].

Japan is one of the world’s largest importers and users of asbestos [16, 17], and the number of deaths due to MPM reached 1500 in 2015 [18]. A total of 100,000 deaths are expected in Japan in the next 40 years [19]. Previous research has demonstrated that patients with MPM in Japan exhibit different care needs in the different stages of the disease. A previous study reported a lack of information about their disease and treatment options upon diagnosis, pain and deteriorated physical condition after extrapleural pneumonectomy, uncomfortable symptoms from chemotherapy, shock of the recurrence of the disease, uncontrolled symptoms in the terminal stage, anxiety and anger about developing disease due to asbestos, and burden of legal procedures in all stages [20]. Nurses who care for patients with MPM also experienced difficulties, such as struggling with care, failure to introduce palliative care, limited support for patients with decision making, difficulty in dealing with families, unsuccessful communication, and emotional distress after being with patients with MPM [21]. Previous studies indicate that people with MPM have a poor QOL. Moore et al. reported that support groups can provide an important source of information and support for patients with MPM and their family members [22]. However, information about the QOL of people with MPM in Japan is anecdotal.

This study investigated the QOL of patients with MPM in Japan and determined factors that correlated with their QOL.

## Methods

### Study design

This was a cross-sectional study. The inclusion criteria were 1) patients who were diagnosed with MPM and 2) those who could respond to self-administered questionnaires written in Japanese. No exclusion criteria were applied. A request for cooperation was sent to all hospitals designated to promote quality oncologic care by the Japanese Ministry of Health and Welfare. Based on their agreements, the questionnaires were sent to the hospitals and distributed to patients with MPM. Questionnaires were also sent to 15 branches of a patient advocacy group (Patients and Family Support Group in Japan) for distribution to survivors of MPM. Completed questionnaires were returned to the researchers by postal mail.

### QOL assessment

A self-administered questionnaire was developed that consisted of 64 questions about QOL and collected information about the patients’ age, gender, duration of their disease, and treatments received. The questionnaire also asked whether the patient had received worker’s compensation or support from the asbestos-related health damage relief system and whether the patient had contact with a patient advocacy group. QOL was assessed with the European Organization for Research and Treatment of Cancer questionnaire (EORTC-QLQ C30; QLQ) [23] and the short version of the core domains of the Comprehensive Quality of Life Outcome questionnaire (CoQoLo) [24]. These measures were included in the distributed questionnaire.

The QLQ is a validated, patient-rated, core questionnaire for assessing the health-related QOL of cancer patients. The questionnaire incorporates 5 functional scales (physical, role, cognitive, emotional, and social), symptom scales (fatigue, pain, and nausea and vomiting), a global health and QOL scale, and single items for assessing additional symptoms commonly reported by cancer patients (i.e., dyspnea, loss of appetite, sleep disturbances, constipation, and diarrhea) as well as the perceived functional influence of the disease and its treatment. All items are scored on a 4-point Likert scale (1 = not at all, 2 = a little, 3 = quite a bit, 4 = very much) except for the global health and QOL scale, which uses a modified 7-point linear analog scale (from 1 = very poor to 7 = excellent). The scores for each scale and single-item measures were averaged and linearly transformed into a score ranging from 0 to 100. A high score for the Global Health Status/QOL represents health-related QOL, whereas high scores for the functional and symptom scales and single items represent worse functional ability or significant symptomatology.

The CoQoLo consists of 10 subscales and 28 items and has been validated for Japanese cancer patients. The CoQoLo assesses the QOL of patients with advanced cancer in the terminal stage to support a ‘good death’ based on the patient’s perspective [24]. In the current study, we applied the short version of the CoQoLo (short CoQoLo) to minimize the burden on participants. The short CoQoLo includes the following 10 items that assess physical and psychological comfort: staying in the patient’s favorite place, maintaining hope and pleasure, good relationships with the medical staff, not being a burden to others, good relationships with family, independence, environmental comfort, being respected as an individual, and having a fulfilling life. These items were answered on a 7-point Likert scale (from 1 = completely disagree to 7 = completely agree).

### Statistical analysis

The scores on the QLQ were calculated using a previously described scoring procedure [25]. The Likert scales for each item on the short CoQoLo were used to score each item. A multiple regression analysis was assessed to estimate the correlations between the QOL scores and the clinical and social factors that potentially affected the factors for the QOL scores. Age was categorized as less than 60 years, 60–69 years, 70–79 years, and 80 years or older. Sex, receiving surgery, receiving chemotherapy, receiving radiotherapy, receiving supportive care, receiving compensation, and membership in an advocacy group were treated as dichotomous variables. The years from diagnosis were divided into categories of less than 2 years and two or more years. A *p* value less than 0.05 was considered statistically significant. The statistical analyses were performed using STATA version 14.2 (STATA corporation, College Station, TX, USA).

## Results

### Collection of questionnaires

Requests for cooperation were sent to 422 cancer hospitals, and 64 (15.2%) agreed to participate. The main reason for nonparticipation was the absence of patients with MPM. In February 2016, 438 questionnaires were distributed throughout the hospitals to patients with MPM. By the end of April 2016, 88 patients had returned the questionnaires to the researchers by postal mail. Additionally, 94 questionnaires were mailed to survivors of MPM through a patient advocacy group in March 2016. Among these, 45 (47.9%) were returned. In total, 133 questionnaires were collected.

### Characteristics of the participants

The characteristics of the participants are presented in Table 1. Overall, 83.5% were male, and the mean age was 69.3 years. The mean (± standard deviation) duration of MPM was 31.0 (± 43.6) months, 55.6% of the patients had undergone surgery, 83.5% had received chemotherapy, 28.6% had received radiotherapy, and 45.9% had received palliative care. Either worker’s compensation or assistance from the asbestos-related health damage relief system was received by 74.4%, and 36.8% were members of a patient advocacy group.

**Table 1:** Sociodemographics of the participants

	N (133)	Percent
Sex		
Male	111	83.5
Female	22	16.5
Age		
≤ 59	17	12.8
60–69	56	42.1
70–79	47	35.3
≥ 80	13	9.8
Duration of disease (months)		
0–11	49	36.8
12–23	35	26.3
24–35	17	12.8
36–47	6	4.5
48–60	6	4.5
≥ 61	20	15.0
Performance status		
0	19	14.3
1	66	49.6
2	21	15.8
3	25	18.8
4	2	1.5
Received treatment	0	0
Surgery	57	55.6
Extra pleural pneumonectomy	31	
Pleurectomy decortication	23	
Unknown	3	
Chemotherapy	111	83.5
Radiotherapy	38	28.6
Palliative Care	61	45.9
Compensated (there is some overlap)	99	74.4
Workmen’s accident compensation insurance	58	
The asbestos-related health damage relief system	61	
Patient and family support group membership	49	36.8

### QOL assessment in MPM survivors

The QOL scores are presented in Table 2. The mean global QOL score was 47.9, and the mean scores for the 5 functional scales, i.e., physical, role, cognitive, emotional, and social function, were 64.4, 54.1, 64.5, 70.1, and 67.0, respectively. Regarding the symptom scales, the mean scores for fatigue, pain, nausea and vomiting, dyspnea, appetite loss, sleep disturbance, constipation and diarrhea were 50.8, 34.7, 12.9, 50.1, 38.3, 36.1, 38.1, and 14.8, respectively. The scores on the symptom scales and functional scales were significantly worse among those with poor performance statuses (PSs).

**Table 2:** Quality of life scores of the survivors with MPM

EORTC QLQ C-30	Mean	SD	Short CoQoLo	Mean	SD
Global QOL	47.9	24.9	Total score	48.9	9.7
Physical functioning	64.4	25.8	Being free from physical pain	3.8	1.9
Role functioning	54.1	30.3	Being able to stay at one’s favorite place	5.3	1.4
Emotional functioning	70.1	24.8	Having some pleasure in daily life	4.4	1.7
Cognitive functioning	64.5	25.7	Trusting physician	5.8	1.5
Social functioning	67.0	28	Feeling like the cause of trouble for others	4.0	1.8
Fatigue	50.8	26.4	Spending enough time with one’s family	5.0	1.6
Nausea & Vomiting	12.9	21.7	Being dependent in daily activities	5.4	1.6
Pain	34.7	29.0	Living in calm circumstances	5.4	1.4
Dyspnea	50.1	29.0	Being valued as a person	5.4	1.3
Insomnia	36.1	30.9	Feeling that one’s life was complete	4.4	1.7
Appetite loss	38.3	34.7			
Constipation	38.1	34.6			
Diarrhea	14.8	23.0			
Financial difficulties	33.1	31.9			

The results of the short CoQoLo assessment revealed favorable scores for ‘Trusting physician’ (5.8), ‘Being dependent in daily activities’ (5.4), ‘Being valued as a person’ (5.4), ‘Being able to stay at one's favorite place’ (5.3), and ‘Spending enough time with one's family’ (5.0). The scores were not very favorable for ‘Having some pleasure in daily life’ (4.4), ‘Feeling that one's life was complete’ (4.4), ‘Feeling like the cause of trouble for others’ (4.0), and ‘Being free from physical pain’ (3.8). The mean total score across the 10 items was 48.9.

### Clinical factors correlated with QOL

The correlations between the QOL scores and the clinical factors are presented in Table 3. The score for the global health status on the QLQ among female survivors was 10.89 points higher than that among males. Long-term survivors (≥ 2 years from diagnosis) and survivors with poor PSs were significantly correlated with poor global health status. The total score on the core domain of the short CoQoLo was also significantly lower among the long-term survivors and survivors with poor PSs.

**Table 3:** Multiple regression analysis of the QLQ-C30 and CoQoLo scores

		QLQ-C30; Global health status			CoQoLo; Core domain total		
		Coefficient	95% CI	*p*-value	Coefficient	95% CI	*p*-value
Age at survey							
	−59	0			0		
	60–69	−3.08	−14.48, 8.33	0.594	2.44	−2.69, 7.56	0.348
	70–79	−4.55	−16.52, 7.43	0.454	2.25	−3.13, 7.63	0.409
	80-	1.10	−14.83, 17.03	0.891	4.97	− 2.19, 12.13	0.172
Sex							
	Male	0			0		
	Female	10.89	1.30, 20.48	0.026	4.03	−0.28, 8.34	0.067
Years from diagnosis							
	< 2	0			0		
	≥ 2	−10.36	−18.53, −2.19	0.011	−4.73	−8.40, − 1.06	0.012
Treatment							
	Surgery						
	(−)	0			0		
	(+)	4.99	−3.30, 13.27	0.235	1.10	−2.62, 4.82	0.558
Chemotherapy							
	(−)	0			0		
	(+)	−5.75	−15.50, 4.00	0.245	0.65	−3.73, 5.04	0.768
Radiation							
	(−)	0			0		
	(+)	1.25	−2.56, 5.06	0.65	1.25	−2.56, 5.06	0.65
Palliative care							
	(−)	0			0		
	(+)	−2.63	−5.93, 0.66	0.116	−2.63	−5.92, 0.66	0.116
Performance Status							
	0	0			0		
	1	−16.55	−27.26, −5.84	0.003	−3.54	−8.36, 1.26	0.147
	2	−34.49	−47.69, −21.28	0.000	−7.64	−13.57, − 1.71	0.012
	3	−40.97	−53.78, −28.15	0.000	−11.42	−17.18, − 5.67	0.000
	4	−73.01	− 102.99, − 43.02	0.000	−24.09	− 37.56, −10.62	0.001
Compensation							
	Not approved	0			0		
	Approved	5.86	−2.98, 14.70	0.192	1.75	−2.22, 5.72	0.385
Patient advocacy group							
	Non-member	0			0		
	Member	0.97	−7.47, 9.41	0.821	0.15	−3.64, 3.95	0.936

## Discussion

In this cross-sectional study, we intended to clarify the QOL of survivors with MPM at various stages of their disease, including diagnosis and during and after cancer treatment. To our knowledge, this is the first study in Japan to focus on the assessment of QOL of patients with MPM and to include a considerable number of long-term survivors.

The QLQ assessment in the current study indicated that emotional function and social function were relatively impaired in survivors of MPM, and the survivors complained more frequently of fatigue and dyspnea. Arber et al. reported that patients with MPM receive insufficient psychososial support at the time of the diagnosis [26]. Previous reports on the QOL of patients with MPM during systemic chemotherapy revealed impairments on QOL scales that were similar to the results reported here [10]. Another study that included patients with MPM who were treated with either chemotherapy or best supportive care produced consistent impairments of QOL [27]. Although the current study included subjects with poorer PSs, the results are quite similar to those of the previous studies and support the notion that patients with MPM experience diverse, overlapping symptoms that are often difficult to control [21, 28]. The QLQ scores reported in the current study were similar to those reported in previous studies of patients with MPM in other countries [10, 11].

The short CoQoLo assessment revealed relatively favorable scores concerning items such as ‘Trusting physician’, ‘Being dependent in daily activities’, ‘Being valued as a person’, ‘Being able to stay at one's favorite place’, and ‘Spending enough time with one's family’. However, the score for ‘Being free from physical pain’ was not favorable, which suggests that pain is an important element of QOL in patients with MPM.

The results of the multiple regression analysis of the QLQ indicated that a longer duration from diagnosis and a poor PS were factors correlated with impaired QOL. The results of multivariate regression analysis of the short CoQoLo scores also indicated that impaired QOL was correlated with poor PS and a longer duration from the diagnosis. A better QOL in patients with better PSs has been widely reported in previous studies [10, 11, 29]. The current study includes a considerable number of people who had survived for more than 2 years. We speculate that MPM can be cured in only a few cases; therefore, a prolonged clinical course would result in more severe and continuous struggles with the disease.

Several studies have been performed from a qualitative perspective, focusing on the MPM patient’s perspective, suggesting that patients living with MPM undergo a traumatic shock and a “Damocles’ syndrome” characterized by intense fears of death and anxiety along with the awareness of the absence of effective treatments [30]. There are other studies performed with semi-structured interviews, those seem to suggest that the reduced QOL of these cancer patients is strictly related with the severity of symptoms, the poor prognosis, along with the awareness of the “unnatural” origin of MPM, the ethical issues connected to the human responsibilities in the contamination, and intense worries for the beloved ones who will survive [14, 26, 31, 32]. From a clinical point of view of the subjective experience of patients living with the disease, specific tailored psychological interventions should be developed in the understanding of the in-depth psychological functioning of these patients.

This study has some limitations. First, the current study included a small convenience sample. We recruited as many patients as possible from the hospitals that provide oncological care and through a patient advocacy group in Japan. Our results may not be representative of the general population of patients with MPM; however, our participants may at least be representative of survivors to a certain extent. Second, our participants had a relatively longer duration of disease, had received surgery, chemotherapy, and/or palliative care, and had better PSs. The data from the patients in the terminal stage and from those with poor general conditions may have been missed due mainly to the difficulty of accessing such people. The QOL of our participants might be better than those of the general population of patients with MPM, which indicates that the QOL of patients with MPM on site may be more impaired. Finally, this study was a cross-sectional study of prevalent cases. A longitudinal study of incident cases is warranted to identify the factors that affect the QOL of incident cases of MPM and to develop systems for the desired support and care.

## Conclusions

Survivors of MPM have impaired function, experience a variety of symptoms, and have a lower QOL. The duration of disease and a poor PS correlated with impaired QOL. Survivors of MPM, even those in good physical condition, need broader support.

## Abbreviations

CoQoLo: Comprehensive quality of life outcome
EORTC: European Organization for Research and Treatment of Cancer
MPM: Malignant pleural mesothelioma
PS: Performance status
QOL: Quality of life
WHO: World Health Organization
